# Knowledge, attitude and preventative practice of tuberculosis in rural communities of Dikgale, Mamabolo and Mothiba health and demographic surveillance system in Limpopo province, South Africa

**DOI:** 10.1186/s12889-023-15845-y

**Published:** 2023-09-01

**Authors:** Ngwanamohuba Mologadi Seloma, Marema Ephraim Makgatho, Eric Maimela

**Affiliations:** 1 Department of Pathology and Medical Sciences, Faculty of Health Sciences, University of Limpopo, Sovenga, South Africa; 2 Department of Public Health and Health Promotion, Faculty of Health Sciences, University of Limpopo, Sovenga, South Africa

**Keywords:** Tuberculosis, Knowledge, Attitude, Practices, Educational interventions

## Abstract

**Background:**

Tuberculosis continues to be a serious public health issue. To reduce the transmission of the disease, it is imperative to address the major obstacle of inadequate understanding regarding the causes, risk factors, treatments, and prevention of pulmonary TB. The study assessed knowledge, attitude, and preventative practices of tuberculosis among community members in Dikgale, Mamabolo and Mothiba (DIMAMO) Health Demographic Surveillance system, Limpopo Province South Africa.

**Methods:**

A cross-sectional clinic-based survey involving 360 participants was conducted at clinics at Dikgale, Mamabolo and Mothiba (DIMAMO) Health and Demographic Surveillance System. A standardised questionnaire on socio-demographic, knowledge, attitude and preventative practices towards tuberculosis based on (WHO) KAP-TB template guidelines was used to collect data. Descriptive statistics on Statistical Package for Social Sciences (SPSS) version 27.0 was used to analyse data.

**Results:**

The results of the cross-sectional survey on KAP-TB illustrated that the participants have good knowledge, attitude, and perception of TB. Majority of the participants (n = 270, 75%) had good general knowledge, while (n = 90, 25%) had poor knowledge about TB. However, the study reports (n = 57, 15.6%) having knowledge of causative agents of TB. Participants showed a favourable attitude toward people who are infected with TB. 87% showed a favourable attitude while only 12.46% showed an unfavourable attitude towards TB. Participants showed a good practice of (71.7%) while (28.3%) of participants had poor practice towards TB.

**Conclusion:**

Health education interventions programme on TB needs to be intensified among the community members to improve TB awareness and reduce transmission. Focused educational interventions on TB aetiology and mode of transmission are required to increase TB preventative practices and improve health-seeking behaviour among community members.

## Background

Tuberculosis is an infectious disease caused by the *Mycobacterium tuberculosis* complex and is one of the leading causes of morbidity and mortality in the world. The most typical type of tuberculosis (TB) presentation is pulmonary tuberculosis (PTB), however, the disease can also affect other tissues and organs, including the lymph nodes, abdomen, and meninges [1–4]. Eight of the 30 high TB burden nations, including India (26%), China (8.5%), Indonesia (8.4%), the Philippines (6.0%), Pakistan (5.8%), Nigeria (4.6%), Bangladesh (3.6%), and South Africa (3.3%), contributed for 86% of all estimated incident cases worldwide [4]. South Africa ranked 14 on the thirty high burden TB/HIV co-infection and multidrug-resistant tuberculosis (WHO) trends list [4].

Tuberculosis is distributed unequally and concentrates in socioeconomically disadvantaged and marginalized demographic groups [5]. The decline of TB in developed countries with better living conditions shows that poor living conditions, poor healthcare-seeking behaviour, and inadequate health systems, favour the spread of TB and the occurrence of the disease [6, 7]. Population in underprivileged communities frequently lacks knowledge of Tuberculosis infections, which results in delayed seeking medical care since they are unaware of the signs and symptoms of TB and preventative practices. Lack of knowledge also contributes to poor treatment outcomes and subsequent disease transmission [5, 8]. The Sustainable Development Goals (SDGs) state that early TB diagnosis, treatment initiation and involvement of Stakeholders, government and communities are crucial in order to reach End TB targets [9–12].

According to research, biomedical measures alone are inadequate to stop the spread of TB and the emergence of drug-resistant TB strains. According to research, increases in knowledge and attitudes as well as changes in socioeconomic level help to prevent and control TB. Educational interventions have also been prioritized by WHO. One of the six fundamental elements of the WHO’s End TB Strategy is raising community knowledge about TB in general and encouraging community involvement in disease control [13]. Studies have shown an association between TB awareness, care seeking, and treatment adherence. To address these challenges, the level of knowledge necessary to develop effective interventional programs in specific areas should be known [3, 14].

The Limpopo Province had the greatest number of patients diagnosed with tuberculosis in 2015/2016, with 96.7% starting treatment according to the health system trust report [15]. Tuberculosis was reported as the fourth highest cause of mortality in Limpopo Province [16–18]. Tuberculosis was reported as the third highest cause of mortality in the Capricorn district, the municipal region of Dikgale, Mamabolo and Mothiba (DIMAMO) Health Demographic surveillance System (HDSS) formerly known as Dikgale Health and Demographic Surveillance System [19].

The DIMAMO HDSS gathers longitudinal population data on life-changing events, health, and socioeconomic factors in order to track lifestyle changes, research non-communicable diseases in rural South Africa, and inform health programs. In hopes of saving lives via greater understanding and focused interventions [20–22]. Though there have been studies on communities’ knowledge, attitudes, and preventive practices (KAP) on tuberculosis in South Africa none have been done at Dikgale, Mamabolo and Mothiba rural communities. The study assessed knowledge, attitude, and preventative practices of tuberculosis among community members in Dikgale, Mamabolo and Mothiba (DIMAMO) Health and Demographic Surveillance system (HDSS), Limpopo Province South Africa.

## Methods

### Study design

The research design was cross-sectional. A standardized KAP-TB questionnaire was used to assess knowledge (causes and mode of transmission), attitude and preventive practice of TB from participants presenting at DIMAMO HDSS community clinics in the Capricorn District of Limpopo Province, South Africa.

### Study setting

The study was conducted at local clinics in DIMAMO HDSS, Capricorn District, Limpopo Province. The DIMAMO HDSS is made up of 15 villages that are adjacent to one another, with 7200 dwellings and a population of around 36 000 people [19]. Tuberculosis is reported as the third highest causes of mortality in the Capricorn district, municipal region of DIMAMO HDSS rural villages formerly known as Dikgale Health and Demographic Surveillance System [19]. The municipality is in Capricorn district, the economic hub in Limpopo Province.

### Study participants

Patients that are 18 years and above that presented at the clinic on the day of data collection for various medical services were invited to participate. Unemployment and illiteracy are rampant among DIMAMO populace.

### Sample size and sampling technique

From the 15 villages in DIMAMO HDSS 8 active primary health care clinics were selected using simple random sampling to participate in the study. To recruit an equal number of participants and non-response rate from the eight clinics that provide health services at DIMAMO HDSS the sample size was rounded up to 360. Forty-five participants were recruited from each clinic.

### Sample size

#### Sample size phase two section

A single population proportion formula was used to calculate the sample size.

$${\rm{N}}\,{\rm{ = }}\,{{\rm{Z}}^{\rm{2}}}\,{\rm{*}}\,{\rm{P}}\,{\rm{*}}\,\left( {{\rm{1 - }}\,{\rm{P}}} \right){\rm{/}}{{\rm{E}}^{\rm{2}}}\,\left( {\rm{2}} \right).$$

From the above-mentioned formula: N is the sample size of the study. Z^2^ is the standard normal variant. P is the expected proportion of population based on previous TB report. E^2^ is the absolute error or precision.

$$\begin{array}{l}{\rm{N}}\,{\rm{ = }}\,{{\rm{Z}}^{\rm{2}}}{\rm{*}}\,{\rm{P}}\,{\rm{*}}\left( {{\rm{1 - }}\,{\rm{P}}} \right){\rm{/}}{{\rm{E}}^{\rm{2}}}\\\,\,\,\,\,{\rm{ = }}\,{\left( {{\rm{1}}{\rm{.96}}} \right)^{\rm{2}}}{\rm{X}}\,{\rm{0}}{\rm{.301}}\left( {{\rm{1}}\,{\rm{ - }}\,{\rm{0}}{\rm{.301}}} \right){\rm{/}}\,{\left( {{\rm{0}}{\rm{.05}}} \right)^{\rm{2}}}\\\,\,\,\,\,{\rm{ = }}\,{\rm{3}}{\rm{.8416}}\,{\rm{x}}\,{\rm{0}}{\rm{.301x}}\,\left( {{\rm{1}}\,{\rm{ - }}\,{\rm{301}}} \right){\rm{/}}\,{\left( {{\rm{0}}{\rm{.05}}} \right)^{\rm{2}}}\\\,\,\,\,\,{\rm{ =  323}}\end{array}$$

$$\begin{array}{l}{\rm{Attrition}}\,{\rm{ = }}\,{\rm{N}}\,{\rm{X}}\,{\rm{10\% }}\,\left( {{\rm{non - response}}\,{\rm{rate}}} \right)\\\,\,\,\,\,\,\,\,\,\,\,\,\,\,\,\,\,\,\,{\rm{ = }}\,{\rm{323}}\,{\rm{X}}\,{\rm{0}}{\rm{.1}}\\\,\,\,\,\,\,\,\,\,\,\,\,\,\,\,\,\,\,\,{\rm{ = }}\,{\rm{32}}{\rm{.3}}\end{array}$$

$$\begin{array}{l}{\rm{Total}}\,{\rm{Sample}}\,{\rm{N}}\,{\rm{ + }}\,{\rm{AT}}\\\,\,\,\,\,\,\,\,\,\,{\rm{ = }}\,{\rm{323}}\,{\rm{ + }}\,{\rm{32}}{\rm{.3}}\\\,\,\,\,\,\,\,\,\,\,{\rm{ = }}\,{\rm{Rounded}}\,{\rm{off}}\,{\rm{to}}\,{\rm{360}}\\\,\,\,\,\,\,\,\,\,\,{\rm{ = }}\,{\rm{45}}\,{\rm{participants}}\,{\rm{were}}\,{\rm{recruited}}\,{\rm{from}}\,{\rm{each}}\,{\rm{clinic}}\end{array}$$

#### Inclusion criteria

The cross-sectional survey included community members that presented at clinics who were 18 years above and gave consent to participants in the study. According to South African law, an 18-year-old person is regarded as an adult with the legal capacity to act independently without a guardian or parent’s permission. Community members that presented to the clinics on the scheduled day of data collection were invited to participate in the study.

#### Exclusion criteria

Community members that are mentally ill or extremely sick and those that did not consent to participate in the study were excluded from participation in the study.

### Data collection procedure

A standardized KAP-TB questionnaire that includes questions on the sociodemographic characteristics of the study participants and their knowledge, attitudes, and practices regarding TB was used to collect the data. The questionnaire was initially created in English using a structured KAP-TB questionnaire adapted from World Health Organization and Stop TB Partnership’s template guidance to create knowledge, attitude, and practice surveys [23]. The questionnaire was then translated into Sepedi, Xitsonga and Tshivenda (the local languages of the study area). Nursing students that speaks Sepedi, Xitsonga and Venda completed the KAP-TB questionnaire for researchers to check comprehensibility after the translation to local languages. The questionnaire was translated back into English using each local language. Corrections were made on the questionnaire to maintain language consistency. Prior to actual data collection the KAP-TB questionnaire was piloted at the DIMAMO HDSS clinic and the participants were not included in the main study.

### Validity

The questionnaire was examined by qualified researchers for content validity, and their feedback was taken into account. The KAP-TB questionnaire was piloted at the targeted demographic area before the actual data collection in order to ensure validity reliability, clarity, and acceptability in the region. The lessons learned from the pilot study helped to validate and further improve the questionnaire.

For reliability, Cronbach’s alpha was 0.618 for the questionnaire, indicating an acceptable level of consistency. Observations of the pilot study were not included in the final analysis.

#### Data collection procedure

On the scheduled day for data collections, patients that presented at the healthcare clinics for various health services were informed of the purpose of the study and methodology in the clinics waiting area. The researcher provided patients with sufficient detailed information on the study so that they can make an informed rational decision to volunteer to participate in the study. Participants who were willing to participate in the study were given informant consent form before completion of the KAP-TB questionnaires. The researcher explained as stated in the consent form participation is voluntary and they may withdraw at any time without providing reasons and this will not interfere with regular care or treatments at the healthcare clinic. The researcher and trained data collector offered assistance to anyone who could not read and write or experienced difficulties on completing the questionnaire. The questionnaires were thoroughly reviewed by the researcher, data collectors and under supervision of the community engagement officer during data collection. To maintain the quality of the data, the principal investigators double-checked the completed questionnaire. Data was collected from March 2021 to May 2021 by researcher and trained data collectors supervised by Community engagement officer.

### Data analysis

Data were cleaned and checked for errors, entered into an excel spreadsheet and analysed using SPSS Version 27.0. Demographic characteristic of participants were summarized into frequency and percentage using descriptive statistic. Statistical significance was determined at a p-value of less than 0.05. The study participant’s overall Knowledge, attitude and practices toward TB were analysed as follows. The participants with knowledge scores greater than or equal to the mean value were considered to have good knowledge. Respondents with a favourable attitude had a score greater than or equal to the mean value of the attitude evaluating questions. Participants with a score more than or equal to the mean value of the preventative practice evaluating questions were considered to have good practices. Binary logistic regression and chi-square test were used to investigate factors associated with knowledge, attitude and preventative practices of TB among participants. Odds ratio (OR) with 95% confidence intervals was used to report the association between independent and dependent variables.

## Results

Table 1 presents the socio-demographic characteristics of study participants. A total of 360 participants were recruited for the study, male (n = 69, 19.2%) participants were lower than female while female participants (n = 291, 80.2%) were majority in the study. The participants in the study were above the age of 18 years. About 40.8% of respondent belonging to the age group of 18–29 years, followed by age group of 30–39 with 21.4%, 20.3% of age group of 40 − 39 while 17.5% belonged to above 60 years. All the participants were black (100.0%) in their colour (Table 1). Regarding their education level, 69% of the participants (n = 250, 69%) had secondary level education, (n = 60, 16.7%) had tertiary education, (n = 35, 9.7%) had primary education and (n = 15, 4.2%) were illiterate. In terms of employment 15% of the participants were employed, 3.6% self-employed and 70% were unemployed and 11.4% were pensioners (Table 1)

**Table 1:** Socio-demographic characteristics of study participants

Characteristics	Frequency	Percentage (%)
**Sex**		
Male	69	19.2
Female	291	80.8
**Age range**		
18–29	147	40.8
30–39	77	21.4
40–59	73	20.3
60	63	17.5
**Race**		
Black	360	100
**Educational level**		
Primary	35	9.7
Secondary	250	69.4
College and universities	60	16.7
No school	15	4.2
**Employment**		
Employed	54	15.0
Self-employed	13	3.6
Unemployed	252	70.0
Pensioner	41	11.4

Table 2 presents participants’ general knowledge on TB. Majority of the participants have heard of TB (n = 301, 83.6%) and only (n = 59, 16.4%) have not heard of TB (Table 2). The main source of information about TB was health worker (n = 150, 41.7%), while (n = 76, 21.1%) heard it from media (Radio, TV), school (n = 49, 13.9%), friend and families (n = 60, 16.7%), and church (n = 21, 5.8%). The knowledge on the causes of TB was very low, majorities of the respondent (n = 141, 39.2%) answered smoking and alcohol, while other responses were poor hygiene (n = 24, 6.7%), bacteria (n = 56, 15.6%), dust and air pollution (n = 94, 26.1%). The participants reported coughing and sneezing droplets (n = 316, 87.8%) as mode of transmission, while others response was (n = 22, 6.1%), sexual intercourse (n = 6, 1.7%) and blood (n = 16, 4.4%) (Table 2). Coughing for more than two weeks were the most common TB symptoms mentioned by the participants (n = 256, 71.1%), followed by weight loss (n = 29, 8.06%), fever (n = 123, 3.33%), night sweats (n = 43, 11.94%), diarrhoea (n = 5, 1.4%) and other (n = 15, 4.2%).

Participants reported moderate knowledge on tuberculosis prevention methods: BCG vaccine (n = 86, 23.89%), covering mouth when cough or sneezing (n = 166, 46.11%), hand wash (n = 20, 5.56%), and ventilation (n = 42, 11.67%), don’t know (n = 10, 2.78%) and other (n = 36, 10.0%) (Table 2).

**Table 2:** Study participants’ general knowledge of TB

Variables	Frequency	Percentage (%)
**Have you heard about TB**		
Yes	301	83.6
No	59	16.4
**Source of information**		
TV	26	7.2
Radio	50	13.9
Health worker	150	41.7
Church	21	5.8
School	49	13.6
Billboard	2	0.6
Family/friend	60	16.7
Other	2	0.6
**Causes of TB**		
Poor hygiene	24	6.7
Smoking/alcohol	141	39.2
Dust/air pollution	94	26.1
Bacteria	56	15.6
Not sure	43	11.9
Other	2	0.6
**TB transmission mode**		
Coughing droplets	316	87.8
Blood	16	4.4
Sexual intercourse	6	1.7
Other	22	6.1
**TB signs and symptoms**		
Cough for 2 weeks or more	256	71.1
Weight loss	29	8.06
Fever	12	3.33
Night sweats	43	11.94
Diarrhoea	5	1.4
Other	15	4.2
**TB prevention methods**		
BCG Vaccination	86	23.89
Covering mouth and nose when coughing	166	46.11
Hand washing	20	5.56
Ventilation	42	11.67
Don’t know	10	2.78
Other	36	10.0

Table 3 presents participants’ attitudes toward TB. The participants show a good attitude toward TB, with (n = 350, 97.2%) of the participants having serious thoughts about TB disease, somewhat serious (n = 7, 1.9%) and not serious (n = 3, 0.8%). 55% (n = 197, 54.7%) thinks they can be infected with TB while (n = 163, 45.3%) thought they cannot be infected with TB. Regarding the reaction to TB, the majority of the participants will visit a health facility if they have signs and symptoms of TB (Table 3). The response showed that 69.9% will visit a health facility, 1.4% will show fear and 0.8% will show shame and sadness. Participants reaction towards people with TB is compassionate with desire to help (n = 34, 94.7%) with (n = 17, 4.7%) having negative attitude towards people with TB. A number of people (n = 307, 85.3%) will support people with TB in their community and few people (n = 9, 2.5%) will reject and (n = 15, 4.2%) will avoid people with TB (Table 3).

**Table 3:** Study participants’ attitude toward TB

Variables	Frequency	Percentage (%)
**Thoughts on seriousness of TB diseases**		
Very serious	350	97.2
Somewhat serious	7	1.9
Not serious	3	0.8
**Do you think you can be infected with TB**		
Yes	197	54.7
No	163	45.3
**Reaction if you had TB symptoms**		
Fear	5	1.4
Shame	3	0.8
Sadness/hopelessness	3	0.8
Visit health facility	349	69.9
**What is your reactions towards people who have TB**		
Compassionate with desire to help	341	94.7
Compassionate but avoid them	17	4.7
No particular feeling	2	0.6
**How does your community treat a person with TB?**		
People are friendly	29	8.1
Reject them	9	2.5
Support them	307	85.3
Avoid them	15	4.2

Table 4 presents participants’ perceptions and preventative practices toward TB. The majority of the participants opened their house windows in the morning (n = 200, 55%) and (n = 84, 23.3%) opened their windows regularly. A limited number of participants open their windows always (n = 54, 15%) and only (n = 22, 6.1%) opened their windows when it is hot. In this study (n = 286, 79.4%) participants opened windows in the car while travelling and (n = 74, 20.6%) kept their car windows closed.

**Table 4:** Study participants’ perceptions and preventative practice toward TB

Variables	Frequency	Percentage (%)
**How often do open windows at Home**		
Regularly	84	23.3
Morning	200	55.6
When it is hot only	22	6.1
Always	54	15.0
**Do you open cars windows Car windows when travelling**		
No	74	20.6
Yes	286	79.4
**Have you have been screened/ checked for TB**		
Yes	163	45.3
No	197	54.7
**Is TB curable?**		
Yes	315	87.5
No	45	12.5
**Choice of TB Treatment**		
Modern health	353	98.1
Traditional healer	6	1.7
Don’t know	1	0.3
**How long should TB treatment be taken to be cured?**		
Weeks	24	6.7
Months	203	56.4
Don’t know	133	36.9
**Risk of defaulting from treatment**		
Death	194	53.9
Relapse	60	16.7
No cure	74	20.6
Develop drug-resistant	17	4.7
Do not know	15	4.2

A reasonable number of participants (n = 163, 45.3%) have tested for TB while majority (n = 197, 54.7%) of participants did not screen for TB. 45% responded that TB is curable (Table 4). Regarding TB treatment, the respondent chooses modern health facility (n = 315, 87.5%) over traditional medicine (n = 6, 1.7%) while one respondent said, “Don’t know). An average number of participants (n = 203, 56.4%) of the respondents that TB treatment durations is months, while 24% said weeks while (n = 159, 43.6%) did not know. The risk of defaulting on TB treatment would lead to death was respondent of 53.9%, relapse 16.7%, no cure 20.6% and developing drug-resistant 4.7% (Table 4).

**Fig. 1:**
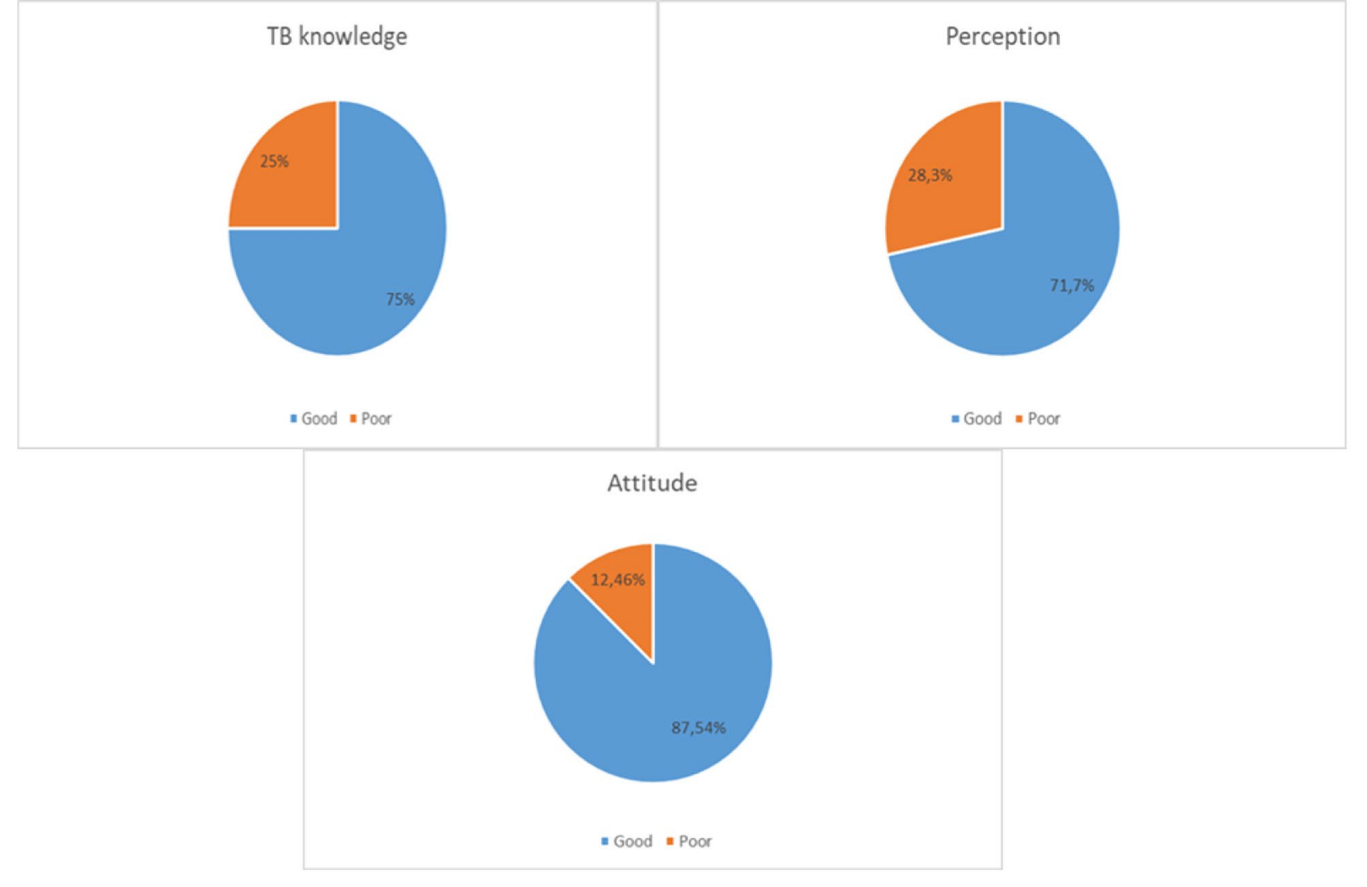
Overall KAP level toward TB

The results of a cross-sectional survey on KAP-TB illustrated that the participants have good knowledge, attitude and perception about TB (Fig. 1). Majority of the participants 75% had good general knowledge, while 25% had poor knowledge about TB. However, only 15.6% of participants had knowledge of bacteria as the cause of TB. Participants also showed a favourable attitude toward people who are infected with TB (Fig. 1). 87% showed a favourable attitude while only 12.46% showed an unfavourable attitude towards TB. Participants showed a good perception of TB with 71.7% while the poor showed 28.3% (Fig. 1).

The results show female gender is associated with good knowledge of TB as compared to male gender (COR 0.167–0.714, 0.004), Age (COR 0.088–0.717, p-0.010) (Table 5). Chi square shows female gender has significant association with good knowledge of TB (Table 5).

**Table 5:** Bivariate analysis of factors associated with participants good knowledge of TB

Variables	B	OR	Cl	Chi square	Fisher’s exact test	P-value
Male	Ref	Ref	Ref	Ref	Ref	Ref
Female	-1.063	0.345	0.167–0.714	7.721	0.009*	0.004*
Age						
30–39	-0.766	0.465	0.190–1.137	3.4556	0.339	0.093
40–59	-1.380	0.252	0.088–0.717			0.010
60–69	-1.138	0.320	0.086–1.195			0.090*
70+	Ref	Ref	Ref	Ref	Ref	Ref
Education level						
Primary	0.447	1.564	0.227–8.837	6.318	0.67	0.612
Secondary	-0.624	0.536	0.096–2.985			0.476
College and university	-1.884	0.152	0.019–1.214			0.076
No school	Ref	Ref	Ref	Ref	Ref	Ref
Socio economic						
Employment	0.143	1.153	0.254–5.232	0.486	0.837	0.853
Self-employed	-0.004	0.996	0.125–7.959			0.997
Employed	-0.058	0.944	0.246–3.616			0.933
Pensioner	Ref	Ref	Ref	Ref	Ref	Ref

B = beta coefficients, OR = Odds ratio, CI = Confidence interval, Ref = Reference, * = significant difference at p < 0.05

## Discussion

The study assessed knowledge, attitude, and preventative practices of tuberculosis among community members in Dikgale, Mamabolo and Mothiba (DIMAMO) Health and Demographic Surveillance system, Limpopo Province South Africa. The findings illustrate that the participants in the present study had good general knowledge, attitude and perception of tuberculosis disease. The findings shows participant’s overall good knowledge of TB is 75% while 25% showed poor knowledge. Although in this study majority of the participants had a good general knowledge of tuberculosis disease, the study reports low awareness of the aetiology of TB. The results are similar to studies conducted by Tolossa et al. [24] and Chinnakali et al. [25] and [1] in Ethiopia, Tamilnadu and India. These studies reported 94.9%, 94% and 92% have heard about the diseases respectively. The majority of the participants in the present study had heard about TB from the health worker, followed by radio and school. The participants reported smoking, alcohol, dust and air pollution as the cause of tuberculosis. Similar misconceptions on the cause of tuberculosis disease were reported in a South African study conducted in Nelson Mandela Bay, Eastern Cape [26]. The results are similar to [1] who reported that the participants in the study reported cold air, hot climate, smoking and food shortages. Studies Tolossa et al. [24] and Chinnakali et al. [25] reported poor awareness about the cause of disease in Sudanese and Ethiopia. TB is a contagious disease with a high frequency in South Africa. As a result, understanding the aetiology, signs, and symptoms of an infectious disease like tuberculosis is critical to limiting its transmission. The results show a need for educational intervention on TB in order to raise more awareness of the disease in rural communities.

The findings of the current study are inconsistent with a study conducted in the rural Eastern Cape, South Africa reported an average of 56% of participants had good knowledge of TB and 44% of participants had poor knowledge of TB [27]. The findings of the current study are higher as compared to the previous studies done in both North Mecha and Thailand. The results by Kasa et al. [3] and Amiri et al. [28] reported that 54% and 62% of the participants had good knowledge about TB, which is lower than the current study. Poor awareness of TB in Thailand and Ethiopia were due to poor knowledge about TB [24, 29]. Similar findings that reported high knowledge of TB (94.9%) among community participants were conducted in Ethiopia Shinile town [24] and (92.8%) in Somalia region of Ethiopia [2].

The Marjory of the participants in this study reported coughing for more than two weeks as the most common answer compared to weight loss, fever, night sweats, and diarrhoea. Knowledge of TB symptoms and transmission techniques has crucial implications for the TB control program in the present investigation area, as well as the Limpopo Province because it could reduce diagnostic and treatment delays, as well as the disease spread. The results are similar to those by Tolossa et al. [24] who reported that 76.7% of participants mentioned coughing for more than two weeks. Most of the participants were aware of prevention methods such as covering the mouth and nose when coughing and only 23.89% of the participants were aware of BCG vaccination protects against TB. The results by Angeline et al. [1] reported a lower number of participants (35.8%) being aware that TB is preventable. The majority of participants responded that covering of mouth and nose when coughing is a preventative strategy was due to most participants were aware of COVID-19 precaution measures and also the study was conducted during the lockdown period. The current NTP relies on passive case findings, patients presenting themselves to health facilities for screening and diagnosis of TB. Lack of knowledge regarding the cause of tuberculosis has a negative impact on preventative methods or health-seeking behaviour. A National TB prevalence study conducted in 2018 reported that nearly two-thirds of those with symptoms suggestive of tuberculosis had not sought treatment at the time of the study, and those who had not sought treatment said the symptoms were minor and hence did not seek medical attention.

Poor knowledge of sign and symptoms may cause a delay in diagnosis and promotes the transmission of tuberculosis among close contacts and the community [1, 2]. The limited knowledge of signs and symptoms of tuberculosis is reported by a study conducted in India where 62.3% of the participant mentioned cough as a symptom of TB and lacked knowledge of the other signs and symptoms [1]. To address inadequate knowledge of the causes, signs and symptoms of TB will require strengthening house contact management interventions in affected populations [5].

The current study participants had a good positive attitude toward TB patients with 87% reporting a favourable attitude while 12.46% showed an unfavourable attitude. The findings are varying with a study conducted in the rural Eastern Cape Province in South Africa, which reported 23.5% of participants to have a good or favourable attitude and 76.5% to have an unfavourable attitude toward TB. The current study participants showed a better attitude toward people infected with TB compared to a study conducted in North Mecha Ethiopia and Thailand. Kasa et al. [3] reported 68% positive attitude in North Mecha and 47.9% positive attitude obtained by [29] in Thailand. The results by Tolossa et al. [24] reported 42.9% of poor attitude and 57.1% of good attitude, among community members in Shinile town Ethiopia. Studies have associated high education level and socioeconomic status with a positive attitude towards TB [1, 27]. Although the unemployment rate was high among the participants in this study majority had secondary education. Interventions such as active case finding within the community and screening of TB patients’ household contacts may benefit from community members’ positive attitudes toward TB. Participants in a study conducted in Mangaung Metropolitan in Free State, South Africa showed a good attitude as a result of health education provided at the primary health care facilities [30].

Negative attitudes and perceptions of TB in the community may affect the social relations of patients afflicted with the disease and hamper TB control programme efforts [24]. Research has found negative perceptions and discrimination of TB patients by society play a role in patients delaying seeking medical care as they fear the stigma associated with TB [3, 27]. The finding of the current study showed that 71.7% of participants had good prevention practices and 28.3% had poor prevention practices for TB. Studies in Iran reported 42.6% and Thailand reported 55.5% of participants to have good preventative practices towards TB, which is low in comparison to the findings in this study [28, 29]. The community’s knowledge of TB forms their perceptions of the disease and affects health-seeking behaviour, adherence to treatment and the success of tuberculosis prevention and control programmes.

In this study female gender and the age group of (40 to 59) showed significant association with good knowledge on TB. The results are in line with Angeline et al. [1] who reported significant association of age and female gender on adequate knowledge. According to a study carried out in Khyber Pakhtunkhwa, Pakistan, and Kabul, Afghanistan women had greater understanding of tuberculosis [31, 32]. The results contradict with two prior investigations found no relationship between participants’ ages and their level of knowledge about TB [33, 34]. Education level and socio-economic showed no statistically associated on good knowledge. The results of the current study are in line with Onyango et al. [26] who reported no statistically significant on independent variables on knowledge.

## Limitation

The KAP-TB questionnaire did not include qualitative questions to allow participants to explain reasons for their attitudes and practices toward TB. The KAP-TB survey included participants that visited clinics on the day of data collection, the results may not be generalised to the district. Despite this limitation, the study gives insight into community members’ knowledge, attitudes, and health-seeking behaviour at the DIMAMO Health and Demographic surveillance. Such data could be useful in informing TB management policy measures.

## Conclusion

The residents of DIMAMO Health and Demographic surveillance System were generally aware of TB. However, they lacked knowledge regarding the causes of TB, as many of the participants believed that smoking or dust were causative agents of TB. As a result, a health education intervention programme aimed at bringing about a significant shift in their knowledge, particularly about the TB causative agent and modes of transmission, prevention, and treatment needs to be intensified among the community members to improve TB awareness.

## Recommendations

The findings from this study provided information that policy makers could use to assess and reshape TB control programs as well as design socioeconomic and public health initiatives to raise awareness of the disease symptoms and improve TB control efforts. Focused educational interventions on TB aetiology and mode of transmission are required to increase TB preventative practices and improve health-seeking behaviour among general community members. To better understand the rationale for delayed treatment seeking and to inform initiatives to close the gap, further qualitative research is needed.

## Data Availability

The datasets generated and analysed during the current study are not publicly available to maintain privacy and ethical restrictions but are available from the corresponding author on reasonable request.

## Abbreviations

BCG: Bacillus Calmette - Guerin
HIV: Human immunodeficiency virus
KAP-TB: Knowledge, Attitude, Perception and Preventative Practices- tuberculosis
PTB: Pulmonary tuberculosis
TB: Tuberculosis
SPSS: Statistical Package Social Sciences
