# Correction: Knowledge, attitude and preventative practice of tuberculosis in rural communities of Dikgale, Mamabolo and Mothiba health and demographic surveillance system in Limpopo province, South Africa

**DOI:** 10.1186/s12889-023-16715-3

**Published:** 2023-09-14

**Authors:** Ngwanamohuba Mologadi Seloma, Marema Ephraim Makgatho, Eric Maimela

**Affiliations:** 1 Department of Pathology and Medical Sciences, Faculty of Health Sciences, University of Limpopo, Sovenga, South Africa; 2 Department of Public Health and Health Promotion, Faculty of Health Sciences, University of Limpopo, Sovenga, South Africa

**Correction: BMC Public Health 23, 1687 (2023)**

**https://doi.org/10.1186/s12889-023-15845-y**

In the original publication of this article [1] the author names were swapped. The correct author names are shown in this correction article, the original article has been updated.
